# Nodular fasciitis growing at the port site of robotic surgery for rectal cancer

**DOI:** 10.1186/s40792-020-01049-8

**Published:** 2020-12-09

**Authors:** Atsushi Yamamoto, Shinji Furuya, Koichi Takiguchi, Makoto Sudo, Katsutoshi Shoda, Hidenori Akaike, Naohiro Hosomura, Yoshihiko Kawaguchi, Hidetake Amemiya, Hiromichi Kawaida, Hiroshi Kono, Daisuke Ichikawa

**Affiliations:** First Department of Surgery, Faculty of Medicine, University of Yamanashi, 1110 Shimokato, Chuo, Yamanashi 409-3898 Japan

**Keywords:** Nodular fasciitis, Port site after robotic surgery, Rectal cancer

## Abstract

**Background:**

Nodular fasciitis (NF) is a type of rare and rapidly growing tumor that affects the muscular fascial layers. Due to its locally aggressive nature and rapid growth, NF can be mistaken as a malignant process on either clinical or histological grounds.

**Case presentation:**

A 61-year-old man was affected by rectal cancer. We performed a robotic, high-anterior resection with lymph node dissection. According to the 8th edition of Union for International Cancer Control, the diagnosis was stage I pT2N0M0. During a routine follow-up 1.5 years after the robotic surgery, a computed tomography examination revealed a tumor in the upper right abdominal wall, at the site of the surgical port, that measured 45 mm. Magnetic resonance imaging indicated a hypo-intensive mass within the right straight muscle of the abdomen. Port site recurrence following the robotic surgery for rectal cancer was suspected, and an ultrasound-guided fine-needle aspiration was performed; it revealed a low-grade myofibroblastic tumor or benign neoplasm, but was inconclusive. We performed an excision of the lesion, and histopathology confirmed NF, seen as a solid, nodular, spindle-cell lesion. The patient was postoperatively followed for more than 1 year without any sign of recurrence of either cancer or NF.

**Conclusions:**

NF is histologically benign, but local recurrence frequently occurs. We encountered a patient with NF at the port site after robotic surgery for rectal cancer.

## Background

Nodular fasciitis (NF), described for the first time by Konwaler et al. [1], is a type of rare and rapidly growing tumor that affects the muscular fascial layers. Due to its locally aggressive nature and rapid growth, NF can be mistaken as a malignant process on either clinical or histological grounds. Despite this, NF can be definitively managed via surgical excision, and recurrence is unlikely. Here, we describe a case of fascial NF, and reference the current understanding on NF’s clinical presentation, radiological and histological features, and management. When considering fast-growing masses, NF must be raised as a potential diagnosis. A misdiagnosis of NF as a malignancy on clinical or histological grounds can lead to an unnecessarily wide excision; in most cases, lesions present by growing rapidly over months, and malignancy may be suspected. On a computed tomography (CT) examination, intra-muscular lesions may not appear to be circumscribed, raising a concern of malignancy. In magnetic resonance imaging (MRI), the features of NF lesions can be non-specific and can look similar to malignant processes, such as rhabdomyosarcoma. On a histological examination, lesions appear to be un-encapsulated and well circumscribed. The lesion and surrounding tissue usually demonstrate a pushing front, although direct infiltration has also been described. For these reasons, an understanding of NF is important when deciding on a surgical excision. On confirmation of NF, an excisional biopsy is both diagnostic and therapeutic, with recurrence unlikely [2].

## Case presentation

**Fig. 1:**
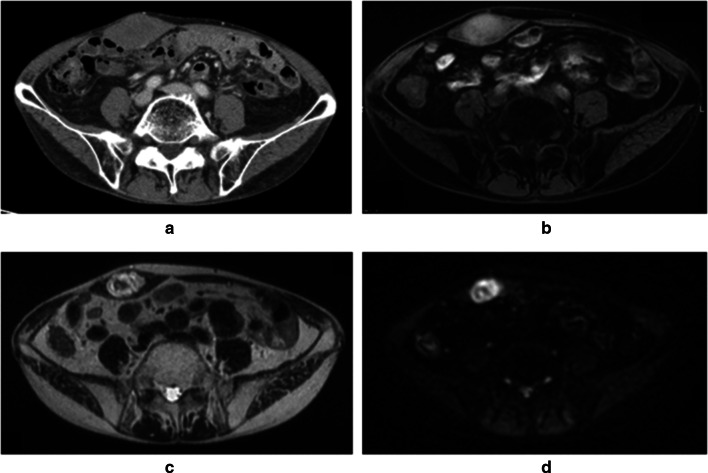
Abdominal CT scan reveals a tumor measuring 45 mm in a diameter at port site in right abdominal wall after 19 months later from the rectal cancer operation (**a**). Abdominal MRI, the mass was hypo-intense to muscle on T1 imaging (**b**), and hyper-intense on T2 (**c**). Some restricted diffusion was seen around the lesion (**d**)

A 61-year-old man was affected by rectal cancer and underwent a robotic, high-anterior resection with lymph node dissection. Histopathologically, well-differentiated adenocarcinoma was suggested. The pathological stage was evaluated as I (pT2N0M0) according to the 8th edition of the Union for International Cancer Control. He presented with a 1-month history of a growing mass in his right abdomen, at the surgical port site, 1.5 years after the robotic surgery. This mass was asymptomatic and non-tender. When first noticed 1 month prior to his complaint, the mass lesion was soft and mobile. He had a medical history of an appendectomy for appendicitis and a procedure for inflammation of the paranasal sinuses. There were no overlying skin changes, the swelling was firm, and the mass was not tethered to the overlying skin. An abdominal CT examination revealed a tumor in the upper right abdominal wall, at the surgical port site, that measured 45 mm (Fig. 1).

An MRI was performed, which showed a solitary lesion measuring 39 mm within the right straight muscle of the abdomen (Fig. 1). The mass was hypo-intense to muscle on T1 imaging, and hyper-intense on T2 imaging. Some restricted diffusion was seen around the lesion. This lesion was believed to represent a malignancy process, such as a recurrence of rectal cancer. Due to the malignancy appearance on both the CT and MRI, a decision was made to perform an excisional biopsy of this lesion.

Due to a suspicion of port site recurrence following the robotic surgery for rectal cancer, an ultrasound-guided, fine-needle aspiration (FNA) was performed. The examination revealed this lesion as either a low-grade myofibroblastic tumor or a benign neoplasm, but was inconclusive.

**Fig. 2:**
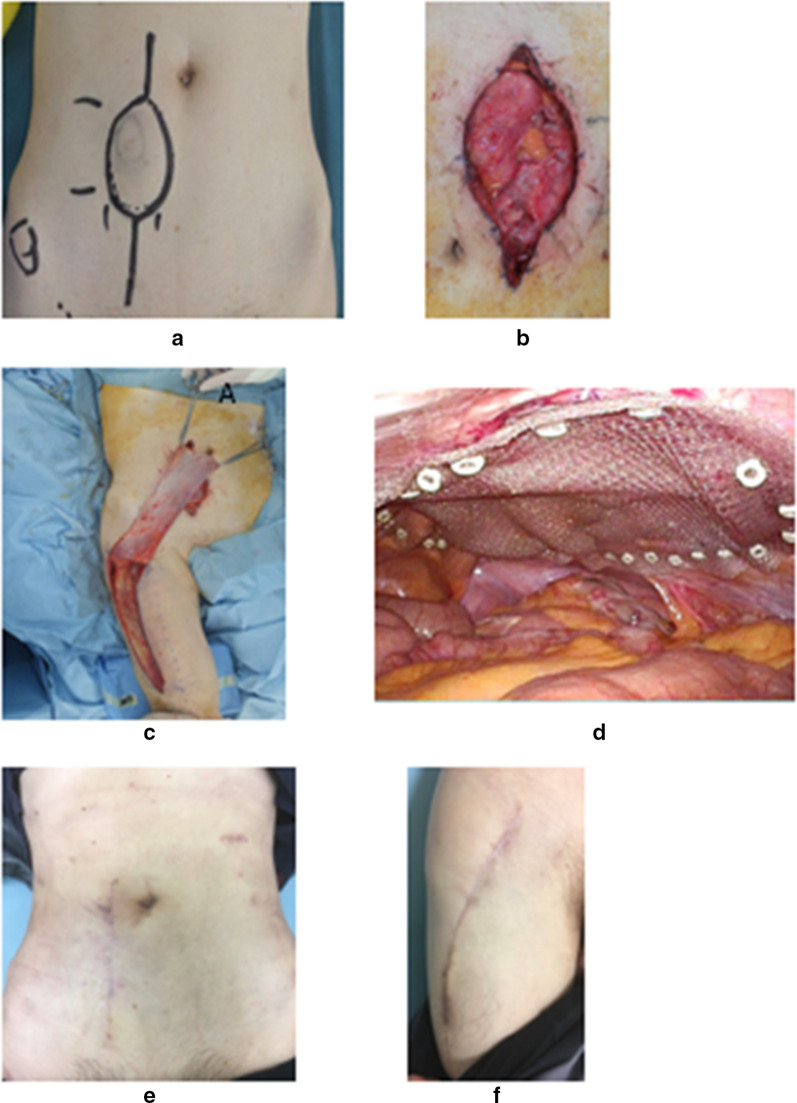
Preoperative findings (**a**). There was no overlying skin changes, the swelling was firm and was not tethered to overlying skin in the upper right abdominal wall. There was the defect of abdominal wall after resection of tumor (**b**). The defect area was covered with mesh as artificial fascia and the right fascia lata with pedicled flap (**c**, **d**). The abdominal and the femoral scar of operation after 3 months (**e**, **f**)

The patient decided to undergo surgery to excise the lesion. Through laparoscopic surgery and a pedicled flap at the right fascia lata, the defect was covered with mesh to create an artificial fascia from the intraperitoneal side (Fig. 2). The patient recovered without any complications at 1 month, and continues to be monitored for recurrence. He has no functional deficits or signs of recurrence.

## Histopathology

**Fig. 3:**
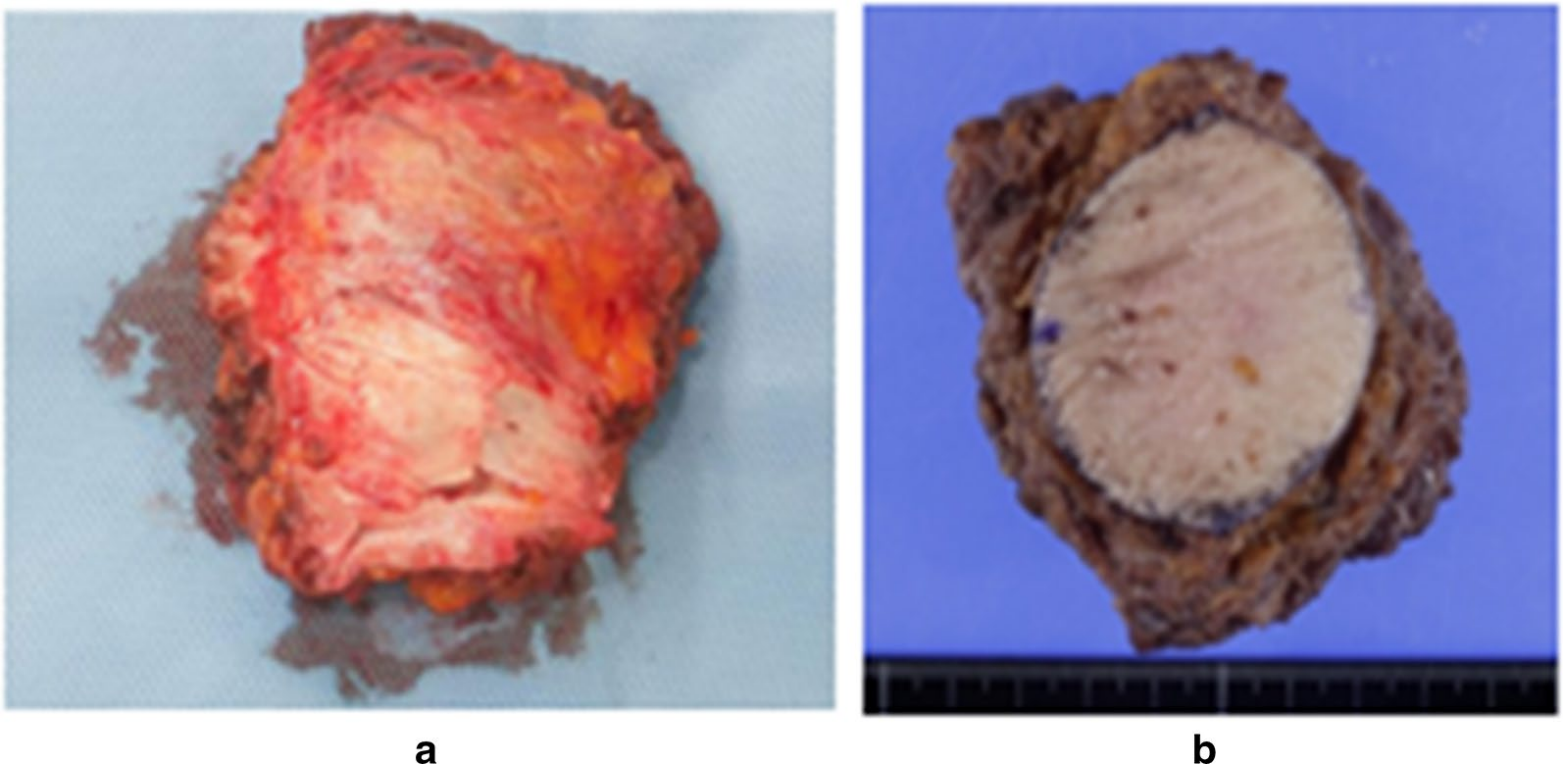
Macroscopic findings of the resected tumor (**a**). Excised specimen (**b**). Well circumscribed solid lesion approximately 40 mm in maximum diameter

**Fig. 4:**
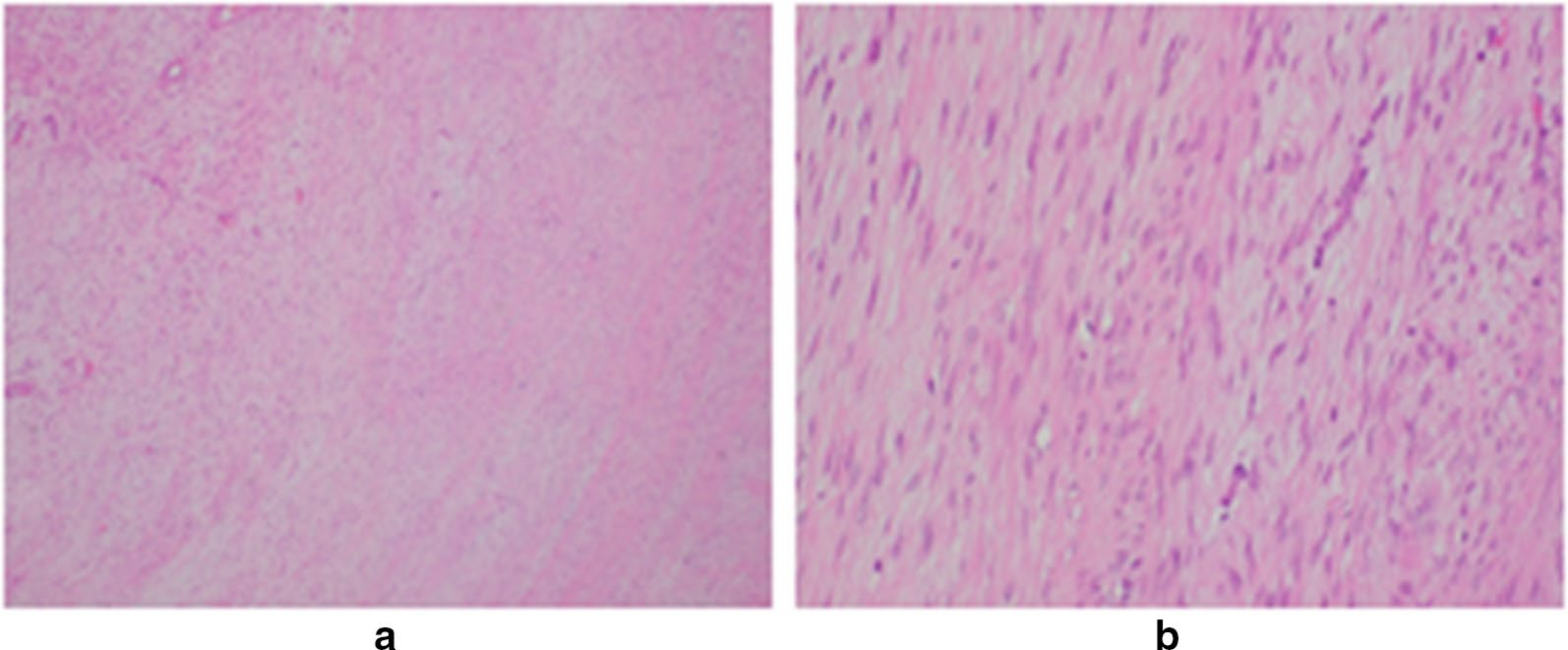
Haematoxylin and eosin stain demonstrating loosely arranged bundles of spindled fibroblastic/myofibroblastic cells without significant atypia, scattered small-sized thin-walled vessels (× 4) (**a**), and extravasated erythrocytes intermingled with spindled myofibroblastic/fibloblastic cells (× 20) (**b**)

**Fig. 5:**
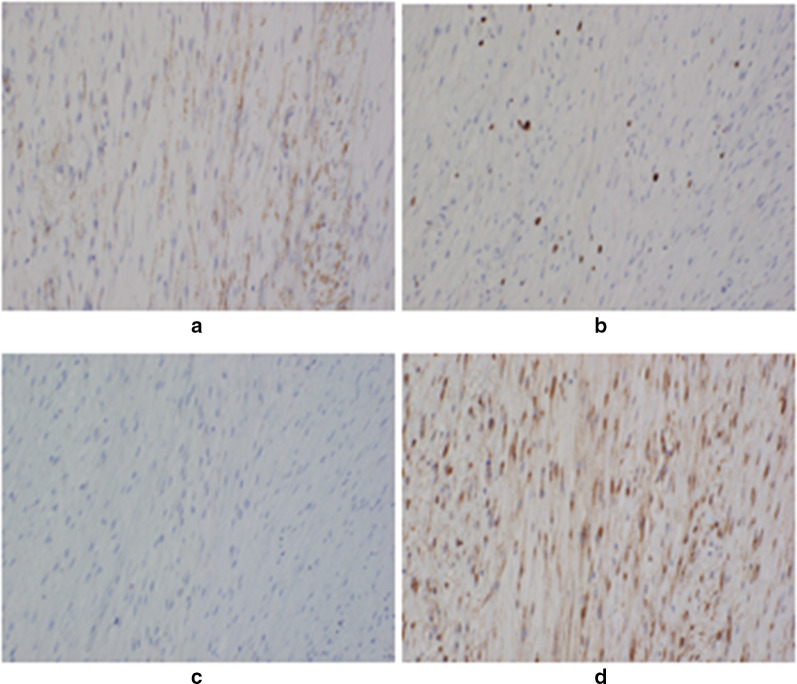
Immunohistochemistry demonstrating positive staining for smooth muscle actin (α-SMA). (× 200) (**a**). Immunohistochemistry demonstrating positive staining at low power (3~5%) for Ki67 (× 200) (**b**). Immunohistochemistry demonstrating negative staining for MUC4 (× 200) (**c**). Immunohistochemistry demonstrating negative staining in the nucleus for β-catenin (× 200) (**d**)

The resected tumor measured 40 × 35 × 30 mm. It was dark red with an unclear border (Fig. 3). There were spindled fibroblastic cells arranged in a herringbone pattern, with hemorrhagia and necrosis in the side of the tumor. Areas of hypercellularity were interspersed with areas of edematous stroma, extravasated erythrocytes, and scattered hemosiderin (Fig. 4). Immunohistochemistry showed that spindled cells had positive staining for α-SMA and negative staining for desmin, S100, CD34, MUC4, and ALK-1 (Fig. 5). Histopathology confirmed nodular fasciitis, seen as a solid, nodular, spindle-cell lesion.

## Discussion

As compared to sarcoma, NF tend to be smaller on diagnosis (less than 40 mm), sharply demarcated (although an infiltrative edge can be seen), with myxoid stroma, and have scant lymphocytic infiltrate with erythrocytes [2]. Furthermore, sarcoma will exhibit cellular pleomorphism and atypia, which should not be seen in NF [3]. Important clinical and histological differential diagnoses of NF include sarcoma [4], fibrosarcoma, fibrous histiocytoma, or myofibromatosis [5]. A relationship between NF and mechanical stimulus has been suggested [1], so NF patients may also describe a history of trauma, although this is not always identified [3]. At first, because the tumor development originated from the surgical wound, port site recurrence was suspected based on a history of rectal cancer. However, possibility for recurrence was supposed to be low considering the diagnosis of the stage of rectal cancer as stage I pT2N0M0. Ultrasound-guided FNA may inform a diagnosis, although FNA results have been inconclusive in many reported cases [3], including ours. The issue of misdiagnoses of NF as other malignant pathologies has also been raised by some authors [6], particularly where NF was incorrectly diagnosed as desmoid-type fibromatosis, committing patients to a more aggressive surgical approach [7]. The final diagnosis is made on histopathology, specifically with immunohistochemical staining of the excised specimen. For example, leiomyosarcomas stain positive for desmin, whereas NF does not [8]. On confirmation of NF, an excisional biopsy is both diagnostic and therapeutic, with recurrence unlikely [3]. Any reported recurrence may indicate either an incomplete excision or the misdiagnosis of a malignant process [4].

## Conclusions

We encountered a patient with NF at the port site after a robotic surgery for rectal cancer. A port site recurrence was suspected, and a resection was performed. NF is histologically benign, but a local relapse frequently occurs.

## Data Availability

All data generated or analyzed during this article are included in this published article.

## Abbreviations

NF: Nodular fasciitis
CT: Computed tomography
MRI: Magnetic resonance imaging
FNA: Fine needle aspiration
UICC: Union for International Cancer Control
